# Seed Biology Updates – Highlights and New Discoveries in Seed Dormancy and Germination Research

**DOI:** 10.3389/fpls.2017.00524

**Published:** 2017-04-11

**Authors:** Hiroyuki Nonogaki

**Affiliations:** Department of Horticulture, Oregon State University, CorvallisOR, USA

**Keywords:** antisense RNA, germination, hormone transport, imprinting, nitrate, nitric oxide

## Abstract

An understanding of the biology of seeds has been greatly advanced in recent years. The progresses, particularly in the field of seed dormancy and germination research, have been made at a remarkable speed. Some of the possible epigenetic mechanisms, including an involvement of non-coding RNA, which were predicted for *DELAY OF GERMINATION1* just a few years ago, have now been demonstrated with strong molecular and genetic evidence. Imprinting, or parent-of-origin-specific gene silencing/expression, which was characterized particularly for developing seeds, was also found in imbibed seeds and suggested for dormancy mechanisms. Hormone biology in seeds, which is the most advanced and almost a traditional area of seed research, also presents a new dimension. Upstream regulators of hormone metabolism and hormone transporters, such as abscisic acid and gibberellin influx/efflux carriers, have been identified. Characterization of the novel posttranslational modification pathways, including the N-end rule and *S*-nitrosylation pathways, which play a critical role in turnover of the major hormone signal transduction proteins, also expanded our knowledge about the complexity of hormone signaling in seeds. These progresses made at the molecular level are significant steps toward a better understanding of how seeds translate soil and other environmental signals into their internal hormone biology and make an important decision to stay dormant or commence with germination.

## Introduction

The previous review article about seed dormancy and germination published in Frontiers in Plant Science (Nonogaki, 2014) summarized the progress made by the international seed research community and highlighted “emerging mechanisms and new hypotheses” at that time. While it has just been a few years since those progresses were reviewed, discoveries continued and more progresses were made, which started to address key biological questions about the mechanisms of seed dormancy and germination. Some of the possible mechanisms predicted by the previous review, such as the regulation of the major seed dormancy genes by long non-coding RNA (lncRNA), have now been demonstrated with convincing evidence. This review will highlight those exciting discoveries to update the current status of our understanding of seed dormancy and germination mechanisms. This article is not intended to provide comprehensive information but will highlight the major discoveries in the relatively unexplored but emerging areas of seed biology research.

## New Players in Nitrate and Nitric Oxide Signaling in Seeds

The molecular mechanisms of seed responses to environmental signals, such as light and temperature, have been well characterized (Bae and Choi, 2008; Toh et al., 2008; Seo et al., 2009; Lim et al., 2013; Barrero et al., 2014; Lee and Choi, 2017). Another signal, which is critical for seeds to sense surrounding environments for germination, is the soil components. Nitrate is a major signal in the soil environment for seeds to detect vegetation gaps and germinate in the desirable spots with the likelihood of successful seedling establishment (Bewley et al., 2013). Nitrate signals received by the maternal plants are integrated with temperature signals during seed development and affect performance of mature seeds (He et al., 2016). Seed responses to nitrate, in terms of dormancy release, are well known, however, the mechanisms of nitrate-responsive gene expression in seeds have been elusive.

**FIGURE 1:**
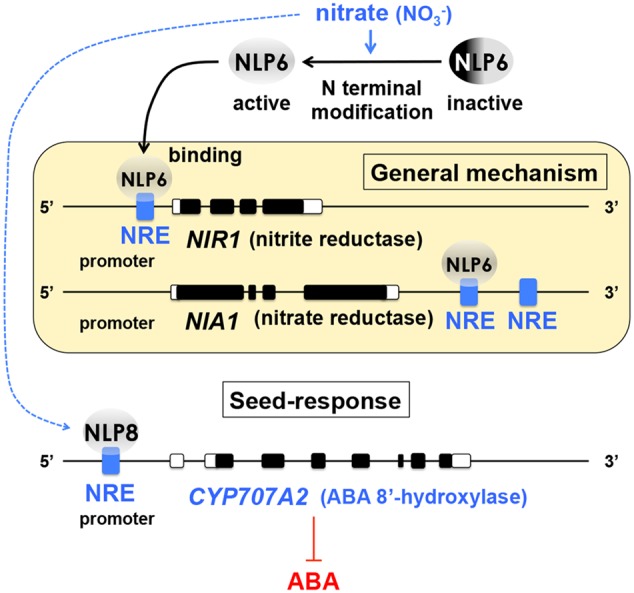
**Nitrate signaling and ABA catabolism in seeds.** An understanding of the general mechanisms of nitrate-induced gene expression in plants has been advanced significantly by the identification of the nitrate responsive *cis*-element (NRE) in the promoter region of *NITRITE REDUCTASE1* (*NIR1*) and the 3′-flanking sequence of the nitrate reductase *NIA1*. Nodule Inception (NIN)-like proteins (NLPs) were identified as NRE-binding factors. NLP6 is activated by nitrate possibly through modification of its N-terminal domain (*top*) and physically interacts with NREs of *NIR1* and *NIA1* (*yellow box*). NLP8 is expressed in a narrow window of Phase I of seed imbibition and directly activates *CYP707A2*, an ABA catabolism gene, which is necessary for seed dormancy release (*bottom*). This discovery has addressed the missing link between nitrate response and ABA metabolism in seeds and advanced our understanding about how the soil environmental signal can be translated into hormone biology in seeds. Based on Konishi and Yanagisawa (2010, 2011, 2013) and Yan et al. (2016).

An understanding of the general mechanisms of nitrate-inducible gene expression in plants was greatly advanced in the past several years. The nitrate-responsive *cis*-element (NRE) was identified in the promoter region of *NITRITE REDUCTASE1* (*NIR1*) (Konishi and Yanagisawa, 2010). The NRE containing promoter enables efficient gene induction in a nitrate-dependent manner (Konishi and Yanagisawa, 2010) and has been tested for nitrate-inducible gene expression in seeds for a technology development purpose (Nonogaki et al., 2015). The nitrate reductase *NIA1*, another nitrate-inducible gene, does not appear to contain NRE in the promoter region, however, the 3′-flanking sequence of the *NIA1* gene, which is downstream of the transcriptional terminator, contains NREs (Konishi and Yanagisawa, 2011) (**Figure 1**). Screening for NRE-binding proteins identified Nodule Inception (NIN)-like proteins (NLPs) as NRE-binding factors (Konishi and Yanagisawa, 2013), which significantly advanced our knowledge on nitrate signaling in plants. NLP6 physically interacts with the NREs in *NIR1* and *NIA1*, most likely upon activation of its N-terminal domain by nitrate (Konishi and Yanagisawa, 2013) (**Figure 1**).

In Arabidopsis seeds, nitrate reduces abscisic acid (ABA) levels during imbibition by upregulating the ABA catabolism gene *CYP707A2* (Matakiadis et al., 2009), which is required for dormancy release (Kushiro et al., 2004). There was a breakthrough in seed biology research, which has revealed that NLP8 is expressed in a very narrow window during Phase I of imbibition and directly binds to NRE in the promoter region of *CYP707A2* to induce its expression (**Figure 1**). In the *nlp8* mutant seeds, both ABA catabolism and germination in response to nitrate are impaired (Yan et al., 2016). This is a significant finding because ABA metabolism is a major determinant of seed germination and therefore identifying its upstream regulators is essential for reaching the core mechanisms of seed dormancy. Factors other than ABA metabolism, such as *DELAY OF GERMINATION1* (*DOG1*) (Bentsink et al., 2006), are also essential for the seed dormancy mechanisms (see below), however, the final “output” of seed dormancy seems to be invariably dependent on expression of ABA biosynthesis genes and concomitant repression of ABA catabolism genes in imbibes seeds (Cadman et al., 2006; Okamoto et al., 2006; Finch-Savage et al., 2007). Application of fluridone, an ABA biosynthesis inhibitor, can induce germination from highly dormant seeds of Arabidopsis Cape Verde Islands (Cvi), from which *DOG1* was identified (Ali-Rachedi et al., 2004). This result also demonstrates the essential role of ABA metabolism in imbibed seeds as the output of the dormancy state. An important biological question is: How are the “fates” (differential expression) of ABA biosynthesis and catabolism genes determined and altered in dormant or non-dormant seeds during early imbibition? Identification of NLP8 as a direct regulator of *CYP707A2* addresses, at least in part, this important question in seed dormancy and germination research. Besides, uncovering NLP8 as the direct link between nitrate and ABA metabolism is also a significant step toward a better understanding of how the soil environmental signals are translated into hormone biology in seeds.

Nitrate could produce nitric oxide (NO), which also stimulates *CYP707A2* expression (Liu et al., 2009; Arc et al., 2013) and seed germination (Bethke et al., 2004, 2007, 2011). However, the NLP8-mediated response is thought to be independent of NO signaling and a direct response to nitrate, because NO-defective mutant seeds still respond to nitrate and germinate in a NLP8-dependent manner (Yan et al., 2016). The nitrate and NO signaling pathways seem to target different transcription factors in seeds.

**FIGURE 2:**
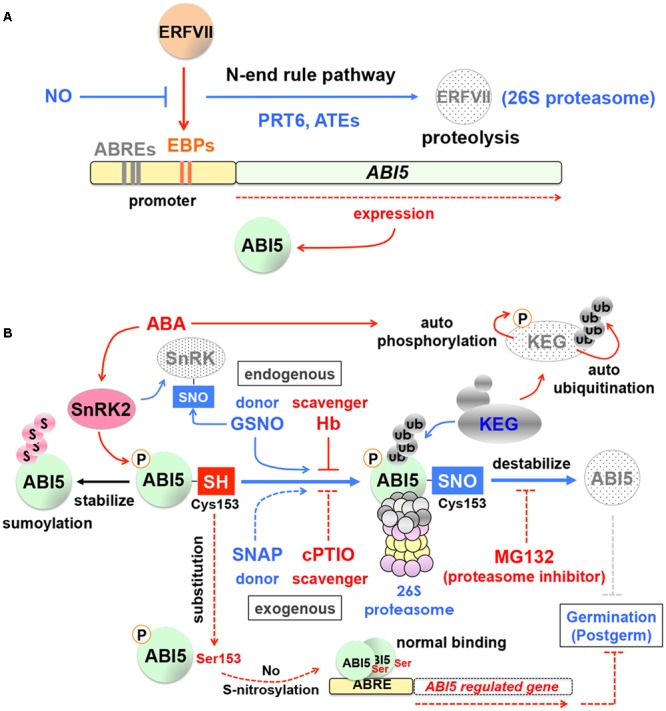
**Nitric oxide (NO) and ABA signaling in seeds. (A)** Indirect regulation of *ABA INSENSITIVE5* (*ABI5*) expression by NO. The group VII ethylene response factors (ERFVIIs) induce expression of *ABI5* through EBP-box *cis-*elements (EBPs) in its promoter region. NO promotes the N-end rule proteolysis pathway and degrade ERFVIIs, thereby reducing *ABI5* expression. ABRE, ABA responsive element; PRT6, PROTEOLYSIS 6; ATE, arginyl-tRNA:protein arginyltransferase. Based on Garzon et al. (2007), Holman et al. (2009) and Gibbs et al. (2014, 2015). **(B**) Direct regulation of ABI5 protein stability by NO. NO counteracts with ABA through the turnover of ABI5 and positively affects seed germination, which is mediated through *S*-nitrosylation. The thiol side chain (-SH) of the cysteine 153 (Cys153) of ABI5 (ABI5-SH) is subject to *S*-nitrosylation by NO, which can be provided by the endogenous donor *S*-nitrosoglutathione (GSNO). In this way, ABI5-SH is converted to ABI5 nitrosothiol (ABI5-SNO), which is destabilized through ubiquitination (ub) by the RING-type E3 ligase KEEP ON GOING (KEG) and degradation by the 26S proteasome pathway. This process reduces ABA signaling and positively affects seed germination and postgermination. In contrast, relatively high levels of ABA promote autophosphorylation of KEG by its own kinase domain (*top-right*), which destabilizes KEG through autoubiquitination and in turn stabilizes ABI5. ABI5 can also be stabilized by sumoylation (s, *top-left*), which prevents ubiquitination, although it makes ABI5 inactive. When Cys 153 of ABI5 is substituted by Ser 153 (*bottom-left*), ABI5 still functions normally, in terms of dimer formation and ABRE binding, however, ABI5 *S*-nitrosylation is abolished, which negatively affects germination and postgermination. Note that SNF1-related protein kinase 2 (SnRK2), which phosphorylates and activates ABI5, is also subject to *S*-nitrosylation (*top-left*). The endogenous NO scavenger hemoglobins (Hb) and the exogenous NO donor *S*-nitroso-*N*-acetyl-DL-penicillamine (SNAP) and scavenger 2-(4-carboxyphenyl)-4,4,5,5 tetramethylimidazoline-1-oxyl-3-oxide (cPTIO) are also shown with the proteasome inhibitor MG132. Based on Bethke et al. (2004, 2007), Perazzolli et al. (2004), Stone et al. (2006), Miura et al. (2009), Liu and Stone (2010, 2014), Ragni et al. (2011), Hill (2012), Albertos et al. (2015) and Wang et al. (2015).

Nitric oxide targets *ABA INSENSITIVE5* (*ABI5*), a major regulator of ABA signaling, which illustrates the crosstalk between the NO and ABA pathways. NO negatively regulates *ABI5* expression by modulating the group VII ethylene response factors (ERFVIIs) through the N-end rule pathway (Gibbs et al., 2014, 2015) (**Figure 2A**). The N-end rule pathway is a ubiquitin-dependent proteolysis pathway, in which N-terminal residues of proteins serve as degradation signals (N-degrons) and determine half-life of proteins (Bachmair et al., 1986; Tasaki and Kwon, 2007; Tasaki et al., 2012). NO destabilizes ERFVIIs, which are upstream regulators of *ABI5*, through the N-end rule and 26S proteasome pathways, thereby suppressing *ABI5* expression (Gibbs et al., 2014, 2015) (**Figure 2A**). In this case, NO regulates *ABI5* at the level of transcription and indirectly through ERFVIIs.

In contrast, a novel pathway, in which NO directly affects ABI5 protein stability, has been identified. While the NO-dependent ERFVII degradation by the N-end rule pathway is mediated through PROTEOLYSIS 6 (PRT6), a RING-type E3 ligase (and arginyl-tRNA:protein arginyltransferase [ATE]) (Holman et al., 2009; Gibbs et al., 2014) (**Figure 2A**), the direct regulation of ABI5 protein by NO is mediated by KEEP ON GOING (KEG), another RING-type E3 ligase. KEG destabilizes ABI5 and acts as a negative regulator of ABA signaling (Stone et al., 2006) (**Figure 2B**). In turn, ABA causes destabilization of KEG through autoubiquitination, which is possibly caused by autophosphorylation of KEG by its own kinase domain (Liu and Stone, 2010) (**Figure 2B**). Therefore, in the presence of relatively high levels of ABA, KEG is unable to remove ABI5, which exerts negative effects on seed germination and postgermination. The regulatory mechanism of ABI5 turnover by KEG was well established, however, it was not known how KEG targets ABI5 for ubiquitination. Phosphorylation plays a critical role for ABI5 activity (Lopez-Molina et al., 2001; Piskurewicz et al., 2008) (**Figure 2B**) and therefore one could speculate that dephosphorylation triggers ubiquitination of ABI5 by KEG. However, phosphorylation status of ABI5 does not affect its turnover by KEG (Liu and Stone, 2014). Thus, it was not known what triggers KEG to target ABI5. This question has been addressed by recent research on the molecular mechanisms of NO-promoted seed germination and seedling growth. It was found that the thiol side chain (-SH) of the cysteine 153 (Cys153) in the ABI5 protein is subject to *S*-nitrosylation by NO, which results in the modified Cys153 with nitrosothiol (ABI5-SNO) (**Figure 2B**) and this modification is the trigger of ABI5 ubiquitination by KEG and its subsequent destabilization by the 26S proteasome pathway (Albertos et al., 2015).

In the native system, NO can be supplied through the endogenous donor *S*-nitrosoglutathione (GSNO) and scavenged by hemoglobins (Hb) (Perazzolli et al., 2004; Hill, 2012; Albertos et al., 2015) (**Figure 2B**). Similar effects to increase or decrease NO can be achieved by applying the NO donor *S*-nitroso-*N*-acetyl-DL-penicillamine (SNAP) or the NO scavenger 2-(4-carboxyphenyl)-4,4,5,5 tetramethylimidazoline-1-oxyl-3-oxide (cPTIO), respectively (**Figure 2B**). Application of the NO donors GSNO and SNAP promotes ABI5 degradation in dormant Arabidopsis seeds (Albertos et al., 2015), providing evidence for NO-dependent ABI5 turnover. Destabilization of ABI5 is prevented by the proteasome inhibitor MG132 despite the presence of NO donors (Albertos et al., 2015), which verifies that NO-dependent ABI5 degradation is mediated through the 26S proteasome pathway (**Figure 2B**).

When a mutation is introduced to the ABI5 protein to substitute Cys153 with Ser153, it does not affect the ABI5 function, such as dimer formation and ABA responsive element (ABRE) binding, however, *S*-nitrosylation and destabilization of ABI5 are abolished by this mutation (Albertos et al., 2015). Thus, ABI5 serves as a NO sensor in seeds and seedlings. Interestingly, SNF1-related protein kinase 2 (SnRK2) that phosphorylates and activates ABI5 is also subject to *S*-nitrosylation (Wang et al., 2015) (**Figure 2B**). It appears that NO antagonizes ABA through more than one layer of regulatory mechanism to promote seed germination and early seedling growth. The native system contains the mechanism to stabilize ABI5 through small ubiquitin-related modifier (SUMO) conjugation (sumoylation), which prevents ubiquitination and degradation (Kerscher et al., 2006; Miura et al., 2007) (**Figure 2B**), although it makes ABI5 inactive (Miura et al., 2009; Liu and Stone, 2014).

## Hormone Transport – Interplay Between Seed Tissues

Hormone levels in seeds are determined mainly by its metabolism – biosynthesis and catabolism. Another critical factor, which could significantly affect hormone responses in seeds, is transport of hormones and its precursors from/to different tissues in a seed. Mapping hormone transport between different tissue domains in the embryonic axis, such as the vascular, cortex and endodermis, is critical for a better understanding of the interplay between the distinct cell layers in the embryo, which generates growth potential for germination. It is conceivable that active hormone transport, rather than (or in addition to) diffusion, is involved in the interaction between the embryo and the endosperm. Our knowledge about the mechanisms of hormone transport in seeds is limited. There is little information about ABA or gibberellin (GA) maxima and gradient in the seed cells during imbibition. Localization of hormone transporters in dormant and germinating seeds needs to be characterized.

There were several breakthroughs in the area of hormone transport in seeds. *Arabidopsis thaliana* ATP-binding cassette (ABC) transporter G family member 25 (AtABCG25), which is a plasma membrane-localized ABA transporter, was found by screening the transposon-tagging lines for mutants exhibiting ABA-sensitivity phenotypes during seed germination and seedling growth (Kuromori et al., 2010). Experiments using isotope-labeled ABA showed that ABA was imported into the AtABCG25-expressing “inside-out” membrane vesicles, which were prepared from insect cells, in an ATP-dependent manner, demonstrating that AtABCG25 is an ABA exporter. When AtABCG25 is overexpressed in plants, it reduces ABA inhibition of seedling growth, which supports the idea that AtABCG25 is an efflux carrier of ABA (Kuromori et al., 2010). A separate study identified AtABCG40 (or Pleiotropic drug resistance transporter 12 [PDR12]) as a plasma membrane-localized ABA transporter, however, in this case AtABCG40 functions as an influx carrier of ABA. Expression of *AtABCG40* in yeast and tobacco BY cells increases their ABA uptake. The mesophyll protoplasts isolated from the *atabcg40* mutants exhibit slower ABA uptake compared to wild type. Consistently, seeds of *atabcg40* exhibit reduced ABA sensitivity in germination (Kang et al., 2010).

**FIGURE 3:**
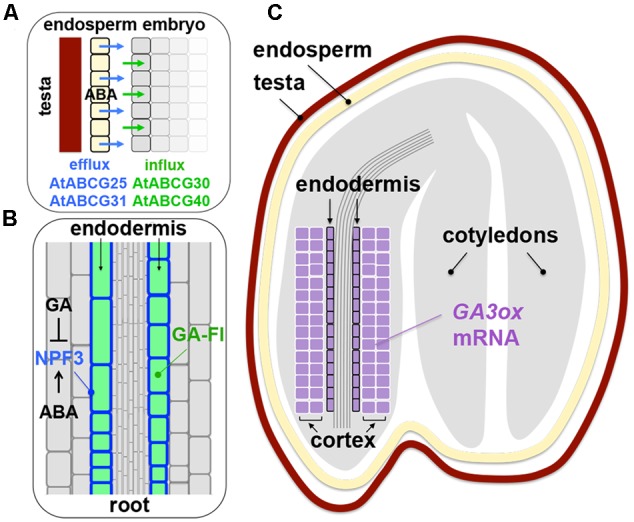
**Hormone transporters in seeds and roots. (A)** Localization of the ABA efflux carriers *Arabidopsis thaliana* ATP-binding cassette (ABC) transporter G family member 25 (AtABCG25) and AtABCG31 (*blue arrows*) in the endosperm (*yellow layer*) and the influx carriers AtABCG30 and AtABCG40 (*green arrows*) in the embryo (*gray layers*) of Arabidopsis seeds, which implies ABA transport from the endosperm to the embryo. Whether the ABA efflux carriers specifically localize at the embryonic side of the plasma membranes of endospermic cells is not known. Details of the localization of the ABA influx carriers in the embryo (e.g., epidermal, endodermal cells) remain to be examined. Based on Kang et al. (2015). **(B)** Localization of the GA influx carrier NITRATE TRANSPORTER1/PEPTIDE TRANSPORTER (NRT1/PTR) family (NPF) member 3 (NPF3) in the plasma membrane of the endodermal cells (*blue*) of Arabidopsis root, which causes accumulation of fluorescently labeled GA (GA-Fl, green) in those cells. *NPF3* expression is reduced by GA and enhanced by ABA. See text for details. Based on Shani et al. (2013) and Tal et al. (2016). **(C)** Localization of *GA3ox* transcripts (*purple*) in the endodermis and cortex of the embryonic axis of Arabidopsis seeds. While little information is available for the localization of GA transporters in seed tissues, it is possible that active transport of GA occurs in a seed also. Based on Yamaguchi et al. (2001).

AtABCG25 exports ABA from the vascular bundles and AtABCG40 imports it to guard cells (Kang et al., 2010; Kuromori et al., 2010, 2014), which is important for translocation of ABA in a plant body. ABA transport has been suggested to occur also in seeds, based on the characterization of AtABCG30 and AtABCG31, which were found to be ABA importer and exporter, respectively. The exporters AtABCG31 and AtABCG25 localize mainly in the endosperm of Arabidopsis seeds while the importers AtABCG30 and AtABCG40 localize mainly in the embryo, suggesting that ABA produced in the endosperm is transported to, and function in, the embryo (Kang et al., 2015; **Figure 3A**). While the hypothesis seems reasonable, there are more questions to be answered. For example, it is not known whether the ABA exporters in the Arabidopsis endosperm specifically localize at the plasma membranes facing the embryo. In the case of auxin transporters PIN-FORMEDs (PINs) in developing embryos, their cellular polarity and the consequential auxin flow in a unidirectional manner have been well documented (Bowman and Floyd, 2008). The endosperm and the embryo in a mature seed are not connected by cells but separated by spatial gaps. Therefore, endosperm cells might just secrete ABA uniformly, which could reach the embryo just by passive diffusion. It is not known whether the ABA importers in the embryo of imbibed seeds localize at the specific cells (e.g., epidermal cells adjacent to the endosperm or inner layers). Are there any specific roles for ABA exported from the endosperm versus ABA produced in the embryo itself? It is conceivable that the endospermic and embryonic origin of ABA target distinct cell layers of the embryo, which can be precisely controlled by specific localization of different ABA transporters, although it is not hard to imagine that the two sources of ABA are inevitably blended in the embryo. ABA production by the endosperm might affect its production in the embryo, or vice versa (i.e., positive feedback or feedforward regulation). Alternatively, ABA exporters might just function to avoid accumulation of ABA in undesirable cell layers by pumping out the hormone from them and the importers might just function to retain ABA in the site of biosynthesis (see below). These strategies could function to contain ABA in specific cell layers, rather than actively transporting the hormone to other locations. Information about the ABA transporters at the cellular level, including an involvement of endocytosis and abiotic stress responses, in roots (not the radicle) is emerging (Park Y. et al., 2016). Similar characterization can be performed to describe the role of ABA transporters in imbibed seeds. While there are still many questions to be answered, the findings about ABCG transporters in seeds suggest an interesting possibility of active transport of ABA between the endosperm and the embryo and opened a new area of seed dormancy and germination research.

In addition to the ABC transporters ABCGs, a different type of ABA transporter, has been identified from the NITRATE TRANSPORTER1/PEPTIDE TRANSPORTER (NRT1/PTR) family (NPF) (Leran et al., 2014). *Arabidopsis thaliana* NPF4.6 (AtNPF4.6), which had been characterized as a low-affinity nitrate transporter NRT1.2, was identified as ABA-IMPORTING TRANSPORTER1 (AIT1) (Kanno et al., 2012). NPF4.6/NRT1.2/AIT1 (called AIT1 hereafter) was found by an elegant screening, which employed a modified yeast two-hybrid system. This system takes advantage of the well-characterized interaction between the ABA receptor and protein phosphatase 2C (PP2C) (Cutler et al., 2010), which occurs only in the presence of a sufficient level of ABA in the cell. The ABA receptor (PYRABACTIN RESISTANCE1 [PYR1]), which is fused to the DNA binding domain (BD-PYR1), and the PP2C (ABA INSENSITIVE1 [ABI1]), which is fused to the activation domain (AD-ABI1), co-induce expression of a reporter (selection marker) gene only when ABA is actively imported into the cell and triggers interaction between the receptor (BD-PYR1) and PP2C (AD-ABI1). Using this system, cDNAs encoding for ABA importers were searched for. The screening identified AIT1 (and other AITs) as a high-affinity, plasma membrane-localized ABA transporter. Overexpression of *AIT1* makes germination of transgenic seeds more sensitive to ABA compared to wild type while *ait1* mutant seeds are insensitive to exogenous ABA (Kanno et al., 2012), both of which support the function of AIT1 as an ABA influx carrier. Detailed localization of AIT1 in imbibed seed cells is not clear. However, the *AIT1* promoter is activated specifically in the vascular tissues of inflorescence stems, which is the same localization as the expression of the major ABA biosynthesis genes. Therefore, it has been proposed that AIT1 function is to maintain the ABA pool size in the site of ABA production (Kanno et al., 2012) (see below for a similar role of the GA influx carrier). It should be noted that AIT1 does not import GA, indole-3-acetic acid (IAA) and jasmonic acid (JA) into the cell, unlike some other NPFs, which transport more than one hormone (Chiba et al., 2015). Thus, the efficient screening of ABA transporters using the ABA receptor as a sensor, further advanced ABA transport studies. More research on the role of active transport of ABA between distinct tissues or its retention by certain cell layers will highlight different functions of seed tissues and cell layers during dormancy.

Information about a GA transporter is also emerging, which may be relevant to ABA transport as well (see below). GA is a mobile hormone, which was clearly demonstrated by grafting experiments (Ragni et al., 2011). Inactive GAs are also subject to long-distance transport (Regnault et al., 2015). Some GA biosynthesis genes, such as *copalyl diphosphate synthase* and *GA 3-oxidase* (*GA3ox*), are expressed in different tissues (provascular vs. cortex/endodermis) in the embryonic axis of Arabidopsis seeds (Yamaguchi et al., 2001). Therefore, it is possible that GA and its precursors are actively transported inside the embryo and a seed by transporters. There is a classical example of GA secretion by cereal embryos to stimulate amylase gene expression in the aleurone layers (Jones and Armstrong, 1971). Likewise, GA produced by the tomato embryo is thought to induce mannanase expression in the endosperm (Groot and Karssen, 1987; Nonogaki et al., 2000; Martinez-Andujar et al., 2012), although involvement of GA transporters in these events has not been demonstrated.

There was a new finding of a GA transporter NPF3 in Arabidopsis. NPF3 has been characterized mainly for roots (not the radicle), however, the findings about the general function of NPF3 in roots provide significant implications for possible roles of GA transporters in seeds. It was known that a fluorescently labeled GA (GA-Fl) accumulates in the endodermal cells in the elongation zone of Arabidopsis roots (Shani et al., 2013). Screening of the T-DNA insertion mutants of the ABC and NPF transporters identified the *npf3* mutants, which were defective in GA-Fl accumulation in the endodermis. NPF3 is an influx carrier, which localizes at the plasma membrane and transports GA-Fl into the root endodermal cells (Tal et al., 2016) (**Figure 3B**). The *npf3* mutants do not show phenotypes in seed germination, probably due to redundancy. *NPF3*-overexpressing seeds exhibit delayed germination (Tal et al., 2016), although more analysis should be performed on the role of NPF3 (and other GA transporters) specifically for *sensu stricto* germination.

*NPF3* expression in roots is repressed by GA and promoted by ABA (Tal et al., 2016) (**Figure 3B**). A plausible interpretation of this transcriptional control is that *NPF3* repression by GA is a negative-feedback mechanism for GA homeostasis, which prevents excessive entry of GA into the cells, while *NPF3* promotion by ABA could be a cell response to increase GA transport into the cells and counteract ABA to maintain a certain GA-ABA balance in the root endodermis. However, the mechanism might not be that simple because interestingly, NPF3 could serve as an ABA importer also; that is, GA and ABA antagonize each other at the level of transport (Tal et al., 2016), in addition to the well-known antagonism at the levels of metabolism and signal transduction (Seo et al., 2009). This is an intriguing mechanism, which has not been explored in terms of hormonal regulation of seed dormancy and germination, and adds a new dimension to GA-ABA antagonism in seeds.

The endodermis (and cortex) is probably the production site of active GA in the embryo during germination, because the transcripts of the rate-limiting GA biosynthesis enzymes GA3ox1 and GA3ox2, which catalyze the final conversion of inactive GAs (GA_20_, GA_9_) to the active forms (GA_1_, GA_4_), are detected in the endodermis (and cortex) (Yamaguchi et al., 2001; **Figure 3C**). It is interesting to examine localization of NPF3 and other GA transporters in the embryonic axis (and the endosperm) of imbibed seeds before testa rupture. If GA importers localize at the endodermis of the elongation zone of the radicle as observed for NPF3 in roots, they probably retain GA in the site of biosynthesis, which is analogous to ABA retention by AIT1 (NPF4.6) in the vascular tissues discussed above. In fact, ectopic expression of *NPF3* in roots with the 35S promoter caused the GA influx carrier to import and trap GA-Fl in the epidermal cells (misguided localization), which caused delayed germination, probably due to reduced transport of GA into the endodermis (targeted growing tissue) (Tal et al., 2016). These results exemplify the importance of precise control of hormone transport and retention in specific tissues and present the complexity of hormonal regulation in roots, which may be applied to seeds also. Comprehensive information about the localization of all ABA and GA metabolism enzymes and transporters (and precursor transporters if any) in different seed tissues will draw a clear picture of hormone production, transport and antagonism during seed dormancy and germination (see EPIGENETICS below for auxin transport).

## Making a Seed Dormancy Gene “Dormant”

Does it make sense to maintain a seed dormancy gene “dormant”? Yes, it does but it seems to be done through antisense. Before entering this topic, it is probably important to touch on the significance of the repression of developmental programs in seeds. A number of lessons have been learned about the biological significance of repression and de-repression of seed developmental programs. During seed development, the embryogenesis program occurs in the embryo proper and part of the suspensor (West and Harada, 1993) while this program is strictly repressed in the rest of suspensor cells. When this control is lost by a mutation, such as *leafy cotyledon1* (*lec1*), aberrant cell divisions occur in the suspensor cells, and a secondary embryo could be formed from these cells, which result in abnormal seeds containing double-embryos (Lotan et al., 1998). These results demonstrate the importance of well-coordinated spatial and temporal repression of certain developmental programs to allow others. Similar repression and de-repression occur also for testa development. The developmental program of the testa, which is precisely repressed in the integuments by the Polycomb Repressive Complex 2 (PRC2) pathway, is de-repressed upon fertilization through auxin signals from the endosperm (Figueiredo et al., 2016). In addition to these examples during seed development, repression and de-repression events play a central role also in the regulation of seed germination. This is well exemplified by the suppression of seed germination by the DELLA proteins during seed dormancy. GA-inducible genes, which are important for seed germination, are repressed by DELLAs (Cao et al., 2006). Upon GA perception by the receptor and its interaction with DELLAs, the repressor proteins are ubiquitinated and subject to degradation or inactivation by the 26S proteasome pathway (McGinnis et al., 2003; Dill et al., 2004; Ueguchi-Tanaka et al., 2005, 2007). In this case, the repression and de-repression at the posttranslational level play a role in the transition from the dormant to germinable state of seeds. The significance of repression of specific transcription factors by small RNAs at the posttranscriptional level has also been demonstrated for hormonal regulation of seed germination (Liu P.-P. et al., 2007; Reyes and Chua, 2007; Nonogaki, 2010).

Seed dormancy itself is a suppressive mechanism, which prevents mature seeds from germinating under conditions otherwise favorable for germination (Bewley et al., 2013). Interestingly, the dormancy mechanisms are also subject to repression when seeds need to become the germinative mode. The previous review focused intensively on the possible epigenetic mechanisms to repress the major dormancy genes (e.g., *DOG1, ABI3*) through chromatin remodeling, such as histone and DNA methylation by the PRC2 and KRYPTONITE (KYP) pathways (Nonogaki, 2014). While there were more developments in research, which reinforced the idea of PRC2 and KYP involvement in *DOG1* regulation (Footitt et al., 2015), this topic will not be repeated here. However, it should be stressed that detailed mechanisms of *DOG1* and *ABI3* silencing by the PRC2 and KYP pathways are still unknown. It is necessary to elucidate the regulatory mechanisms controlling expression of the major dormancy genes, including possible involvement of lncRNAs in their silencing, which was predicted by the previous review (Nonogaki, 2014).

**FIGURE 4:**
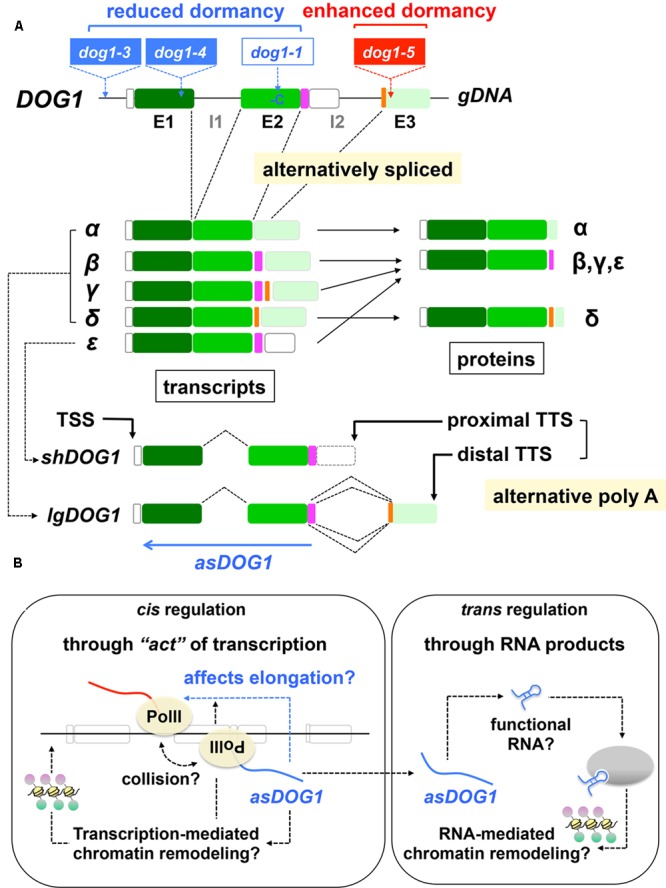
**Regulation of *DOG1* expression and function. (A)** Structures of the *DOG1* gene. *Top, DOG1* gDNA with exons (E1, E2, E3) and introns (I1, I2). Alternatively spliced regions are highlighted in *pink* and *orange*. Approximate positions of the *dog1* mutations (T-DNAs in *dog1-3, dog1-4, dog1-5* and a single-base deletion [-C] in *dog1-1*) are also indicated. *Middle*, alternative *DOG1* transcripts (*α, β, γ, δ, 𝜀*) and the corresponding proteins. Note that *DOG1-𝜀* is not exactly an alternative splicing product. See text for details. *Bottom*, alternatively polyadenylated short *DOG1* (*shDOG1*), which is identical to *DOG1-𝜀* and long (*lgDOG1*) transcripts, which comprises *DOG1-α, -β, -γ* and *-δ*. The transcriptional start (TSS) and termination (TTS) sites are indicated. Approximate position and the orientation of antisense *DOG1* (*asDOG1*) are shown as a *blue arrow*. Based on Bentsink et al. (2006), Dolata et al. (2015), Nakabayashi et al. (2015), Cyrek et al. (2016), Fedak et al. (2016). **(B)** Possible mechanisms of *asDOG1* function. Relatively stable *asDOG1* RNA could function as a regulatory RNA, in a sequence-specific manner or through its secondary structure, for RNA-mediated chromatin remodeling (*right panel, trans* regulation). However, allele-specific *asDOG1* expression has indicated that *asDOG1* functions in *cis* (*left panel*). The “act” of transcription itself, rather than its product (RNA), exerts the negative effects of *asDOG1* expression to *DOG1* expression and dormancy. Antisense expression could cause transcriptional interference and affect transcription elongation, which is known to be important for *DOG1* expression and seed dormancy while transcription-mediated chromatin remodeling is also possible. Based on Shearwin et al. (2005), Hongay et al. (2006), Geisler and Coller (2013), Kornienko et al. (2013), Pelechano and Steinmetz (2013), Fedak et al. (2016), Quinn and Chang (2016).

Information about the triggers of *DOG1* and *ABI3* silencing by the PRC2 and KYP pathways is still missing. However, recent studies on the regulatory mechanisms of *DOG1* expression started to decode how the seed dormancy gene could be repressed. When the *DOG1* gene was first identified, the presence of several splicing variants was reported (Bentsink et al., 2006). Further analysis provided more detailed information about the five transcript variants (*α, β, γ, δ, 𝜀*), which produce three different proteins (Nakabayashi et al., 2015) (**Figure 4A**). Among them, *DOG1-𝜀* is the predominant form in the developing Arabidopsis seeds (Nakabayashi et al., 2015) (although *DOG1-𝜀* is not exactly a splicing variant; see below). One could speculate that alternative splicing differentiates function of proteins, including their subcellular localization and potential to impose dormancy. However, all the three proteins are transported to the nucleus (Nakabayashi et al., 2015), which is critical for the predicted function of DOG1 as a regulatory protein (Nakabayashi et al., 2012). Overexpression analysis suggests that all the three isoforms are functional in terms of seed dormancy induction, although they are more stable when co-expressed. DOG1 is thought to function as a homodimer (in a protein complex) (Nakabayashi et al., 2015). Therefore, formation of heterodimers may not explain the better stability of DOG1 proteins. The mechanisms underlying the positive role of co-expression of DOG1 isoforms for their stability is unknown.

When the Arabidopsis ortholog of the yeast spliceosomal component NineTeen complex-Related proteins 1 (AtNTR1) is mutated, it causes the major defects of intron retention and exon skipping in *DOG1*. This misregulation of *DOG1* splicing reduces seed dormancy, although this phenotype may not be caused by alternative splicing itself but is probably the consequence of reduced *DOG1* expression level in this mutant (Dolata et al., 2015). As focused in the previous review, efficiency of transcription elongation is a critical factor for *DOG1* expression and seed dormancy (Liu Y. et al., 2007; Liu et al., 2011; Mortensen and Grasser, 2014; Nonogaki, 2014). AtNTR1 is thought to control RNA polymerase II (PolII) at the splice sites and serve as the checkpoint of transcription elongation (Dolata et al., 2015).

Another mechanism to produce transcript variants is alternative polyadenylation, which generates transcripts differing in 3′ ends (Di Giammartino et al., 2011; Cyrek et al., 2016). The two forms of *DOG1* transcripts, *short DOG1* (*shDOG1*) and *long DOG1* (*lgDOG1*), which are produced by alternative polyadenylation, have been characterized (Cyrek et al., 2016). *shDOG1* is identical to *DOG1-𝜀* while *lgDOG1* comprises *DOG1-α*, -*β*, -*γ* and -*δ* (**Figure 4A**). The C-terminus of DOG1 protein is absent or not conserved in many plant species, suggesting that it is not essential for the DOG1 function. In fact, *shDOG1* is sufficient to complement the *dog1* mutation and recovers seed dormancy (Cyrek et al., 2016). Although there is some discrepancy in published results in terms of the importance of longer versions of *DOG1* transcripts, there seems to be a consensus that the short two-exonic DOG1 is functional and the major protein that is necessary for seed dormancy (Nakabayashi et al., 2015; Cyrek et al., 2016; Fedak et al., 2016).

There is little conservation of the exon 3 region of *DOG1* genomic DNA in terms of encoded polypeptide sequences. In contrast, this region is highly conserved at the level of DNA, which is extended (back) to intron 2 (Fedak et al., 2016). The conservation of this region of *DOG1* sequence at the DNA level, which is contradictory to the low evolutionary pressure for the protein sequences in the same region, implies a possible role of this genomic region of *DOG1* as a production site of a regulatory ncRNA. In fact, expression of a lncRNA in an antisense orientation (antisense *DOG1* [*asDOG1*]) from this region (and the vicinity) has been found (Fedak et al., 2016) (**Figure 4A**). Its expression is not spurious transcriptional noise but is regulated by a transcriptionally active promoter in an antisense orientation, which has been experimentally verified (Fedak et al., 2016). Expression of *asDOG1* negatively affects expression of *shDOG1*, suggesting that *asDOG1* is a negative regulator of *DOG1* expression and seed dormancy. The mutations in the (sense) *DOG1* promoter (*dog1-3* [T-DNA]), exon 1 (*dog1-4* [T-DNA]) and exon 2 (*dog1-1* [1-bp deletion]) cause reduced or little seed dormancy (Bentsink et al., 2006; Cyrek et al., 2016; Fedak et al., 2016). In contrast, a mutation in the exon 3 (*asDOG1* promoter) region (*dog1-5* [T-DNA]) rather enhances seed dormancy (Cyrek et al., 2016; Fedak et al., 2016) (**Figure 4A**), providing convincing evidence for the role of *asDOG1* as a negative regulator of seed dormancy.

Antisense *DOG1* is a relatively stable RNA (a half-life of approximately 46 min) (Fedak et al., 2016), which is typical of regulatory RNAs, and therefore it is possible that the *asDOG1* function depends on the RNA molecule at the posttranscriptional level. However, the detailed analysis by allele-specific *asDOG1* expression has concluded that *asDOG1* is unable to function in *trans* but does function in *cis* (Fedak et al., 2016). That is, the products of *asDOG1* transcription (RNA molecules themselves) may not be important but the “act” of transcription itself (Kornienko et al., 2013; Pelechano and Steinmetz, 2013) is probably the cause of *DOG1* repression (Fedak et al., 2016). The co-transcriptional effects of antisense expression, rather than posttranscriptional regulation by antisense RNA molecules, are known to cause transcriptional interference (Pelechano and Steinmetz, 2013). Transcriptional interference could be mediated by various mechanisms including direct collision of RNA polymerases and promoter competition (Shearwin et al., 2005; Pelechano and Steinmetz, 2013; Quinn and Chang, 2016) (**Figure 4B**). In yeast, an antisense-mediated transcriptional interference blocks transcription elongation of the *IME4* gene (Hongay et al., 2006; Pelechano and Steinmetz, 2013). Therefore, it is possible that *asDOG1* expression affects transcription elongation of *DOG1*, which has been demonstrated to be a critical factor for seed dormancy (Liu Y. et al., 2007; Liu et al., 2011; Mortensen and Grasser, 2014; Nonogaki, 2014) (**Figure 4B**).

Since *asDOG1* is a repressor of seed dormancy, it is reasonable to hypothesize that *asDOG1* might play a regulatory role in germination induction. However, both *DOG1* and *asDOG1* expression is reduced during seed imbibition, and therefore the *asDOG1* function may be restricted to seed maturation (Fedak et al., 2016). Modification of DOG1 protein function, rather than its transcriptional control, may be critical for germination induction through after-ripening (Nakabayashi et al., 2012; Nee et al., 2016). Expression levels of *asDOG1* during the maturation stage might determine the depth of seed dormancy in mature seeds, although more analyses are necessary to conclude the precise role of this interesting mechanism of *asDOG1* in seed dormancy biology.

Here, only *DOG1*, one of the best-characterized dormancy genes, in terms of alternative splicing, alternative polyadenylation and the role of antisense RNA, was focused on. However, other dormancy genes, such as *ABI3* and its ortholog in wheat *Viviparous 1* (*Vp-1*), also produce transcript variants, which are developmentally regulated and important for dormancy (McKibbin et al., 2002; Sugliani et al., 2010). Therefore, more discoveries are anticipated from extended studies of the biological roles of alternative splicing, alternative polyadenylation and antisense transcription in regulation of other dormancy genes, including those in different species.

## Epigenetics – Updates and New Developments

The topic of epigenetic regulation of seed dormancy and germination, which was intensively covered by the previous review, was bypassed above. However, as witnessed through the *asDOG1* study, novel mechanisms associated with epigenetic regulation are emerging. Therefore, some updates and new developments in epigenetic studies on seed dormancy and germination will be briefly examined here.

**FIGURE 5:**
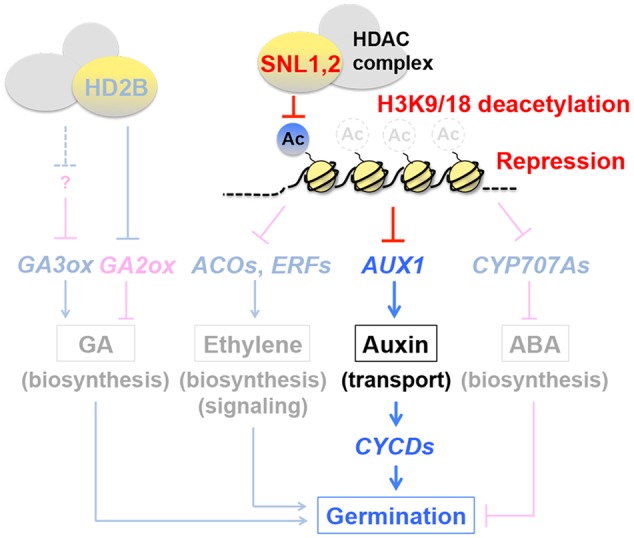
**Regulation of hormone synthesis, signaling and transport in seeds by histone deacetylation.** Previous studies had indicated that histone deacetylation modulates the GA, ethylene and ABA pathways (*shaded background*). HISTONE DEACETYLASE 2B positively affects GA levels in seeds and germination by repressing the GA catabolism gene *GA2ox* and enhancing the GA biosynthesis genes *GA3ox1* and *GA3ox2*. SWI-INDEPENDENT3 (SIN3)-LIKEs (SNLs) in the histone deacetylase (HDAC) complex exert negative effects on ethylene levels in seeds and germination by repressing the ethylene biosynthesis genes *1-AMINOCYCLOPROPANE-1-CARBOXYLATE OXIDASEs* (*ACOs*) and signaling genes *ETHYLENE RESPONSE FACTORs* (*ERFs*), through deacetylation of histone H3 at lysines 9 and 18 (H3K9/18). SNLs repress *CYP707A1* and *CYP707A2* also, which results in ABA accumulation in seeds and negative regulation of germination. A new study has revealed that the negative role of SNLs in seed germination (speed) is also mediated through the auxin pathway. Unlike the other hormonal pathways where metabolism or signaling genes are the targets of histone deacetylation, SNLs repress *AUXIN RESISTANT 1* (*AUX1*), an auxin influx carrier, thereby affecting auxin transport (and synthesis). Repression of *AUX1* reduces the expression of the downstream factors D-type cyclin genes *CYCDs*, which are positive regulators of germination. The auxin transporter appears to play an important role for (the synthesis and) proper distribution of auxin in the radicle tip. Based on Wang et al. (2013, 2016), Yano et al. (2013), Nonogaki (2014).

As summarized in the previous review, deacetylation of histone H3 at lysines 9 and 18 (H3K9/18), which is a repressive mark of gene expression, plays a critical role in regulation of seed germination through the GA, ethylene and ABA pathways (Nonogaki, 2014) (**Figure 5**, shaded background). HISTONE DEACETYLASE 2B (HD2B) promotes GA accumulation in seeds by suppressing the GA catabolism gene *GA2ox2* and (indirectly) enhancing *GA3ox1* and *GA3ox2*, which positively affect germination (Yano et al., 2013). In contrast, SWI-INDEPENDENT3 (SIN3)-LIKEs (SNLs) in the histone deacetylase (HDAC) complex exert negative effects on germination by reducing ethylene levels and signals in seeds through repression of the ethylene biosynthesis genes *1-AMINOCYCLOPROPANE-1-CARBOXYLATE OXIDASEs* (*ACOs*) and signaling genes *ERFs* (Wang et al., 2013). The negative effects of the SNLs on germination are exerted also through repression of the ABA catabolism genes *CYP707A1* and *CYP707A2*, which results in ABA accumulation in seeds (Wang et al., 2013) (**Figure 5**).

The previous findings had revealed the inhibitory roles of SNLs in seed germination through the ethylene and ABA pathways and their antagonism. A new development in research suggests that SNLs exert negative effects on germination through the auxin pathway also. SNL1 and SNL2 repress *AUXIN RESISTANT 1* (*AUX1*) (Maher and Martindale, 1980) through H3K9/18 deacetylation (Wang et al., 2016) (**Figure 5**). Unlike the SNL targets in the ethylene and ABA pathways, which are hormone metabolism and signaling genes, *AUX1* is an auxin influx carrier (Bennett et al., 1996; Yang et al., 2006), suggesting that SNLs modulate seed germination through auxin transport. Here also, the importance of hormone transport in seeds, which was discussed above, is emerging. Auxin could affect seed germination positively and negatively at low (0.03–3 nM) and high (0.3–1 μM) concentrations, respectively (Hsueh and Lou, 1947; Liu P.-P. et al., 2007; He et al., 2012). Application of the auxin synthesis inhibitor aminoethoxyvinylglycine (AVG) and transport inhibitors 2,3,5-triidobenzoid acid (TIBA) and 1-naphthoxyacetic acids (1-NOA) negatively affects germination speed, suggesting that certain levels of auxin synthesis and transport are necessary for normal seed germination. *AUX1* is thought to affect germination through the synthesis and distribution of auxin (possibly at low concentrations) in the radicle tip (Wang et al., 2016). *AUX1* is not essential for radicle emergence, however, it plays a significant role for germination speed through the activation of the D-type cyclin genes *CYCDs* (Wang et al., 2016), which are known to play a role in germination (Masubelele et al., 2005) (**Figure 5**). The function of AUX1 as a transporter in seeds still needs to be investigated into more details. However, the SNL-AUX1 study has made dual impacts on epigenetics and hormone transport in seeds. Seed vigor, including germination speed, is an important aspect of seed quality in agriculture. The SNL-type of regulation of seed germination by epigenetics, which could be affected by seed production conditions and kept as a “memory” in seeds, may be an important constituent of seed vigor. Therefore, this area of research should also be expanded for applied aspects of seed biology.

Another new development of epigenetic research, which is relevant to the seed dormancy and germination mechanisms, is a finding of imprinting in imbibed seeds. Imprinting, or parent-of-origin-specific gene silencing/expression, is known for both animals and plants (Haig and Westoby, 1989, 1991; Moore and Haig, 1991; Feil and Berger, 2007). In imprinting, either the maternal or paternal origin of gene is specifically silenced, which results in preferential expression of the counterpart, independently of Mendelian genetics (Feil and Berger, 2007; Pignatta and Gehring, 2012). The biological significance of imprinting is explained by parental conflicts over resource allocation. Sibling offspring of one mother, which carry genes from different fathers, compete with each other for available resources while the mother favors equal distribution of resources to all offspring. This creates a conflict between the mother and each offspring, which is a “manifestation” of the conflict between the mother and the father (Haig and Westoby, 1989).

**FIGURE 6:**
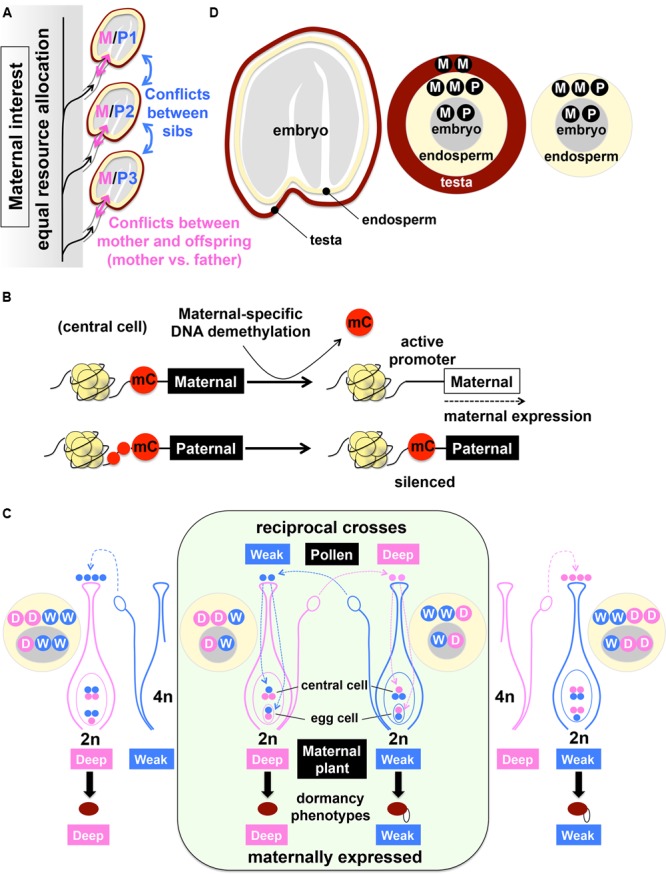
**Imprinting and maternal expression of dormancy traits and genes. (A)** Illustration of parental conflict over resource allocation in developing seeds. Imprinting, or parent-of-origin-specific gene expression, is explained by parental conflict over resource allocation. Each offspring competes with siblings for available resources (*blue double-headed arrows*) while the mother favors equal resource allocation to all offspring (*black arrows*). The conflict between the mother and each offspring (*pink double-headed arrows*) is a “manifestation” of the conflict between the mother (M, maternal genome) and the father (P, paternal genome). Based on Haig and Westoby (1989, 1991). **(B)** Maternal-specific activation of gene expression by DNA demethylation (mC removal) in the endosperm, which results in paternal-specific gene silencing. Based on Kinoshita et al. (2004), Ikeda and Kinoshita (2009), Jullien and Berger (2009). **(C)** Reciprocal crosses between deeply (Deep) and weakly (Weak) dormant accessions, using diploid (2n, *inside green panel*) or tetraploid (4n, *outside green panel*) plants as pollen donors. *Pink* and *blue dots* indicate genomic information and dosage from Deep (D) and Weak (W) dormant accessions, respectively. The F-1 hybrid seeds from reciprocal crosses between D and W tend to phenocopy the maternal seed dormancy traits, which are not explained by dosage effects. The genotypes of the embryo (*gray circle*) and the endosperm (*yellow circle*) are shown. See text for details. Based on Piskurewicz et al. (2016). **(D)** Contribution of the maternal (M) and paternal (P) genome to the embryo (*gray*), endosperm (*yellow*) and testa (*brown*).

The “parental tug-of-war” (Moore and Haig, 1991), between males favoring collective resource acquisition by their own offspring and a female favoring equal resource allocation to all offspring, is exactly what could happen during seed development (**Figure 6A**). Individual seeds compete with each other, which creates the conflicts between the maternal plant and developing seeds. The parent-of-origin effects on seed size, which are also regulated by chromatin remodeling, have been well documented for crossing between plants in different ploidy and the mutants defective in chromatin remodeling (Scott et al., 1998; Ohto et al., 2007). Imprinting during endosperm development has been well characterized (Hsieh et al., 2011; Gehring, 2013; Tonosaki and Kinoshita, 2015). Unlike mammals where *de novo* methylation causes allele-specific gene silencing, DNA methylation and silencing in both alleles are the default state of the imprinted genes in the endosperm (central cell). The allele-specific DNA demethylation, which removes the repressive marks specifically from the maternal allele, drives maternal-specific gene expression in the central cell (Kinoshita et al., 2004; Penterman et al., 2007; Zhu et al., 2007; Jullien and Berger, 2009; Hsieh et al., 2011; Park K. et al., 2016) (**Figure 6B**). While imprinting has been characterized for seed development, it is not clear whether imprinting plays a role in mature seeds, particularly for dormancy and germination.

Recent studies focusing on the molecular mechanisms of maternal expression of seed dormancy traits started to shed light on a possible role of imprinting in seed dormancy. When reciprocal crosses are performed between a deeply dormant accession of Arabidopsis (Cvi) and a relatively weak dormant accession (C24), the two populations of F-1 hybrid seeds from these reciprocal crosses exhibit distinct levels of dormancy, which tend to phenocopy the maternal traits (Piskurewicz et al., 2016): the F-1 seeds produced from the deeply (D) dormant accession as the mother are more dormant compared to those produced from weakly (W) dormant accession as the mother (**Figure 6C**). The genetic information in the embryo, which comes from the maternal (M) and paternal (P) genome (M/P), is essentially the same (D/W or W/D) between the two populations of F-1 seeds (**Figures 6C,D**). The maternally expressed dormancy phenotypes can be caused by the testa, which is derived from the integuments, a maternal (MM) tissue (DD or WW). The maternal effects of testa properties on seed dormancy phenotypes have been well demonstrated by the reciprocal crosses between wild type and *transparent testa* (*tt*) mutants, which exhibit defects in testa pigmentation and dormancy (Debeaujon and Koornneef, 2000; Debeaujon et al., 2000, 2007). However, both Cvi (DD) and C24 (WW) have the pigmented testa. Therefore, it is more likely that the differential expression of dormancy between the reciprocal crosses was caused by the endosperm. The endosperm contains two doses from the maternal genome while the paternal genome contributes a single dose (MM/P) to this tissue (**Figure 6D**). Therefore, the two populations of F-1 seeds differ in their endosperm genotypes (DD/W or WW/D), which could cause dosage effects (**Figure 6C**). However, when this possibility is tested by using tetraploid as pollen donors, which breaks off the genomic imbalance in the endosperm (DD/WW or WW/DD), the maternal expression of the dormancy phenotypes is still observed (Piskurewicz et al., 2016) (**Figure 6C**). Therefore, dosage effects in the endosperm do not explain the maternal expression of dormancy phenotypes. The remaining possibility – imprinting in the endosperm – has been suggested as an alternative explanation (Piskurewicz et al., 2016). Indeed, expression of 71 maternally expressed genes (MEG) and 5 paternally expressed genes (PEG) in the endosperm of dormant seeds and 50 MEG and 8 PEG expression in non-dormant seeds have been detected, with MEG expression showing close correlations with seed dormancy levels (Piskurewicz et al., 2016). Since imprinting occurs during seed development as mentioned above and it could be carried over through dormant seeds, more studies may be necessary to conclude the causal effects (cause or consequence?) of imprinting in imbibed seeds on the maintenance of seed dormancy. Nonetheless, possible involvement of imprinting in seed dormancy and germination regulation is very interesting. Maternal gene expression and histone modification are also emerging as a likely mechanism of heterosis in hybrid seeds (Alonso-Peral et al., 2017; Zhu et al., 2017), which has been a long-lasting question in basic science and is also an important foundation of seed business. Expanding the area of seed epigenetics will advance both basic and applied seed biology.

## Concluding Remarks

This review highlighted a limited number of major findings that started to address the key questions in seed biology, by focusing on the relatively less explored and challenging areas, such as hormone metabolism upstream, hormone transport and lncRNA-mediated gene regulation. There were more findings relevant to hormonal signaling (Jiang et al., 2016), seed structures (Graeber et al., 2014; De Giorgi et al., 2015), crop seed dormancy (Nakamura et al., 2016; Sato et al., 2016; Torada et al., 2016) and many others, which were not integrated into this review due to the limited space and the scope of this article. Different dots and lines provided by other findings will probably be connected to each other by more discoveries in the near future. There are still many questions to be answered. For example, how is *CYP707A2* activated without nitrate in after-ripened seeds? Is it still NLP8-dependent or independent? There are other important questions in an evolutionary context also. How did *DOG1* emerge over the course of evolution and when did *asDOG1* appear and become a *DOG1* repressor? Is imprinting in seed dormancy genes indeed advantageous for plant survival? If so, how is it managed in gymnosperm seeds, which lack the endosperm? What are the crucial differences between megagametophyte vs. endosperm, in terms of gene silencing and chromatin remodeling machinery? These difficult questions will probably be answered by elegant experiments like those highlighted in this review and also by unexpected discoveries in seed and other plant sciences in the future. There are exciting times ahead for seed biology research.

## Author Contributions

The author confirms being the sole contributor of this work and approved it for publication.

## Conflict of Interest Statement

The author declares that the research was conducted in the absence of any commercial or financial relationships that could be construed as a potential conflict of interest.

## References

[B1] Albertos P. Romero-Puertas M. C. Tatematsu K. Mateos I. Sanchez-Vicente I. Nambara E. (2015). S-nitrosylation triggers ABI5 degradation to promote seed germination and seedling growth. *Nat. Comm.* 6:8669 10.1038/ncomms9669 PMC4639896 26493030

[B2] Ali-Rachedi S. Bouinot D. Wagner M. H. Bonnet M. Sotta B. Grappin P. (2004). Changes in endogenous abscisic acid levels during dormancy release and maintenance of mature seeds: studies with the Cape Verde Islands ecotype, the dormant model of *Arabidopsis thaliana*. *Planta* 219 479–488. 10.1007/s00425-004-1251-4 15060827

[B3] Alonso-Peral M. M. Trigueros M. Sherman B. Ying H. Taylor J. M. Peacock W. J. (2017). Patterns of gene expression in developing embryos of Arabidopsis hybrids. *Plant J.* 89 927–939. 10.1111/tpj.13432 27880012

[B4] Arc E. Sechet J. Corbineau F. Rajjou L. Marion-Poll A. (2013). ABA crosstalk with ethylene and nitric oxide in seed dormancy and germination. *Front. Plant Sci.* 4:63 10.3389/fpls.2013.00063 PMC3607800 23531630

[B5] Bachmair A. Finley D. Varshavsky A. (1986). In vivo half-life of a protein is a function of its amino-terminal residue. *Science* 234 179–186. 10.1126/science.3018930 3018930

[B6] Bae G. Choi G. (2008). Decoding of light signals by plant phytochromes and their interacting proteins. *Annu. Rev. Plant Biol.* 59 281–311. 10.1146/annurev.arplant.59.032607.092859 18257712

[B7] Barrero J. M. Downie A. B. Xu Q. Gubler F. (2014). A role for barley CRYPTOCHROME1 in light regulation of grain dormancy and germination. *Plant Cell* 26 1094–1104. 10.1105/tpc.113.121830 24642944 PMC4001371

[B8] Bennett M. J. Marchant A. Green H. G. May S. T. Ward S. P. Millner P. A. (1996). *Arabidopsis AUX1* gene: a permease-like regulator of root gravitropism. *Science* 273 948–950. 10.1126/science.273.5277.948 8688077

[B9] Bentsink L. Jowett J. Hanhart C. J. Koornneef M. (2006). Cloning of *DOG1*, a quantitative trait locus controlling seed dormancy in *Arabidopsis*. *Proc. Natl. Acad. Sci. U.S.A.* 103 17042–17047. 10.1073/pnas.0607877103 17065317 PMC1636575

[B10] Bethke P. C. Gubler F. Jacobsen J. V. Jones R. L. (2004). Dormancy of *Arabidopsis* seeds barley grains can be broken by nitric oxide. *Planta* 219 847–855. 10.1007/s00425-004-1282-x 15133666

[B11] Bethke P. C. Libourel I. G. L. Aoyama N. Chung Y.-Y. Still D. W. Jones R. L. (2007). The Arabidopsis aleurone layer responds to nitric oxide, gibberellin, and abscisic acid and is sufficient and necessary for seed dormancy. *Plant Physiol.* 143 1173–1188. 10.1104/pp.106.093435 17220360 PMC1820924

[B12] Bethke P. C. Libourel I. G. L. Vitecek J. Jones R. L. (2011). “Nitric oxide methods in seed biology,” in *Seed Dormancy: Methods and Protocols* ed. Kermode A. R. (Totowa, NJ: Humana Press) 385–400. 10.1007/978-1-61779-231-1_22 21898267

[B13] Bewley J. D. Bradford K. J. Hilhorst H. W. M. Nonogaki H. (2013). *Seeds: Physiology of Development, Germination and Dormancy*. New York, NY: Springer 10.1007/978-1-4614-4693-4

[B14] Bowman J. L. Floyd S. K. (2008). Patterning and polarity in seed plant shoots. *Annu. Rev. Plant Biol.* 59 67–88. 10.1146/annurev.arplant.57.032905.105356 18031217

[B15] Cadman C. S. C. Toorop P. E. Hilhorst H. W. M. Finch-Savage W. E. (2006). Gene expression profiles of Arabidopsis Cvi seeds during dormancy cycling indicate a common underlying dormancy control mechanism. *Plant J.* 46 805–822. 10.1111/j.1365-313X.2006.02738.x 16709196

[B16] Cao D. Cheng H. Wu W. Soo H. M. Peng J. (2006). Gibberellin mobilizes distinct DELLA-dependent transcriptomes to regulate seed germination and floral development in Arabidopsis. *Plant Physiol.* 142 509–525. 10.1104/pp.106.082289 16920880 PMC1586041

[B17] Chiba Y. Shimizu T. Miyakawa S. Kanno Y. Koshiba T. Kamiya Y. (2015). Identification of *Arabidopsis thaliana* NRT1/PTR FAMILY (NPF) proteins capable of transporting plant hormones. *J. Plant Res.* 128 679–686. 10.1007/s10265-015-0710-2 25801271

[B18] Cutler S. R. Rodriguez P. L. Finkelstein R. R. Abrams S. R. (2010). Abscisic acid: emergence of a core signaling network. *Annu. Rev. Plant Biol.* 61 651–679. 10.1146/annurev-arplant-042809-112122 20192755

[B19] Cyrek M. Fedak H. Ciesielski A. Guo Y. W. Sliwa A. Brzezniak L. (2016). Seed dormancy in Arabidopsis is controlled by alternative polyadenylation of *DOG1*. *Plant Physiol.* 170 947–955. 10.1104/pp.15.01483 26620523 PMC4734566

[B20] De Giorgi J. Piskurewicz U. Loubery S. Utz-Pugin A. Bailly C. Mene-Saffrane L. (2015). An endosperm-associated cuticle is required for Arabidopsis seed viability, dormancy and early control of germination. *PLoS Genet.* 11:e1005708 10.1371/journal.pgen.1005708 PMC4683086 26681322

[B21] Debeaujon I. Koornneef M. (2000). Gibberellin requirement for Arabidopsis seed germination is determined both by testa characteristics and embryonic abscisic acid. *Plant Physiol.* 122 415–424. 10.1104/pp.122.2.415 10677434 PMC58878

[B22] Debeaujon I. Leon-Kloosterziel K. M. Koornneef M. (2000). Influence of the testa on seed dormancy, germination, and longevity in Arabidopsis. *Plant Physiol.* 122 403–414. 10.1104/pp.122.2.403 10677433 PMC58877

[B23] Debeaujon I. Lepiniec L. Pourcel L. Routaboul J. M. (2007). “Seed coat development and dormancy,” in *Seed Development, Dormancy and Germination* eds Bradford K. J. Nonogaki H. (Oxford: Blackwell Publishing) 25–49. 10.1002/9780470988848

[B24] Di Giammartino D. C. Nishida K. Manley J. L. (2011). Mechanisms and consequences of alternative polyadenylation. *Mol. Cell* 43 853–866. 10.1016/j.molcel.2011.08.017 21925375 PMC3194005

[B25] Dill A. Thomas S. G. Hu J. Steber C. M. Sun T.-P. (2004). The Arabidopsis F-box protein SLEEPY1 targets gibberellin signaling repressors for gibberellin-induced degradation. *Plant Cell* 16 1392–1405. 10.1105/tpc.020958 15155881 PMC490034

[B26] Dolata J. Guo Y. W. Kolowerzo A. Smolinski D. Brzyzek G. Jarmolowski A. (2015). NTR1 is required for transcription elongation checkpoints at alternative exons in *Arabidopsis*. *EMBO J.* 34 544–558. 10.15252/embj.201489478 25568310 PMC4331007

[B27] Fedak H. Palusinska M. Krzyczmonik K. Brzezniak L. Yatusevich R. Pietras Z. (2016). Control of seed dormancy in *Arabidopsis* by a *cis*-acting noncoding antisense transcript. *Proc. Natl. Acad. Sci. U.S.A.* 113 E7846–E7855. 10.1073/pnas.1608827113 27856735 PMC5137729

[B28] Feil R. Berger F. (2007). Convergent evolution of genomic imprinting in plants and mammals. *Trends Genet.* 23 192–199. 10.1016/j.tig.2007.02.004 17316885

[B29] Figueiredo D. D. Batista R. A. Roszakt P. J. Hennig L. Kohler C. (2016). Auxin production in the endosperm drives seed coat development in *Arabidopsis*. *eLife* 5:e20542 10.7554/eLife.20542 PMC5135394 27848912

[B30] Finch-Savage W. E. Cadman C. S. C. Toorop P. E. Lynn J. R. Hilhorst H. W. M. (2007). Seed dormancy release in Arabidopsis Cvi by dry after-ripening, low temperature, nitrate and light shows common quantitative patterns of gene expression directed by environmentally specific sensing. *Plant J.* 51 60–78. 10.1111/j.1365-313X.2007.03118.x 17461781

[B31] Footitt S. Muller K. Kermode A. R. Finch-Savage W. E. (2015). Seed dormancy cycling in Arabidopsis: chromatin remodelling and regulation of DOG1 in response to seasonal environmental signals. *Plant J.* 81 413–425. 10.1111/tpj.12735 25439058 PMC4671266

[B32] Garzon M. Eifler K. Faust A. Scheel H. Hofmann K. Koncz C. (2007). *PRT6*/At5g02310 encodes an *Arabidopsis* ubiquitin ligase of the N-end rule pathway with arginine specificity and is not the *CER3* locus. *FEBS Lett.* 581 3189–3196. 10.1016/j.febslet.2007.06.005 17572409

[B33] Gehring M. (2013). Genomic imprinting: insights from plants. *Annu. Rev. Genet.* 47 187–208. 10.1146/annurev-genet-110711-155527 24016190

[B34] Geisler S. Coller J. (2013). RNA in unexpected places: long non-coding RNA functions in diverse cellular contexts. *Nat. Rev. Mol. Cell Biol.* 14 699–712. 10.1038/nrm3679 24105322 PMC4852478

[B35] Gibbs D. J. Conde J. V. Berckhan S. Prasad G. Mendiondo G. M. Holdsworth M. J. (2015). Group VII ethylene response factors coordinate oxygen and nitric oxide signal transduction and stress responses in plants. *Plant Physiol.* 169 23–31. 10.1104/pp.15.00338 25944828 PMC4577381

[B36] Gibbs D. J. Isa N. M. Movahedi M. Lozano-Juste J. Mendiondo G. M. Berckhan S. (2014). Nitric oxide sensing in plants is mediated by proteolytic control of group VII ERF transcription factors. *Mol. Cell* 53 369–379. 10.1016/j.molcel.2013.12.020 24462115 PMC3969242

[B37] Graeber K. Linkies A. Steinbrecher T. Mummenhoff K. Tarkowska D. Tureckova V. (2014). *DELAY OF GERMINATION 1* mediates a conserved coat-dormancy mechanism for the temperature- and gibberellin-dependent control of seed germination. *Proc. Natl. Acad. Sci. U.S.A.* 111 E3571–E3580. 10.1073/pnas.1403851111 25114251 PMC4151772

[B38] Groot S. P. C. Karssen C. M. (1987). Gibberellins regulate seed germination in tomato by endosperm weakening: a study with gibberellin-deficient mutants. *Planta* 171 525–531. 10.1007/BF00392302 24225716

[B39] Haig D. Westoby M. (1989). Parent-specific gene-expression and the triploid endosperm. *Am. Nat.* 134 147–155. 10.1086/284971

[B40] Haig D. Westoby M. (1991). Genomic imprinting in endosperm: its effect on seed development in crosses between species, and between different ploidies of the same species, and its implications for the evolution of apomixis. *Philos. Trans. R. Soc. Lond. B Biol. Sci.* 333 1–13. 10.1098/rstb.1991.0057

[B41] He H. Willems L. A. Batushansky A. Fait A. Hanson J. Nijveen H. (2016). Effects of parental temperature and nitrate on seed performance are reflected by partly overlapping genetic and metabolic pathways. *Plant Cell Physiol.* 57 473–487. 10.1093/pcp/pcv207 26738545

[B42] He J. N. Duan Y. Hua D. P. Fan G. J. Wang L. Liu Y. (2012). DEXH box RNA helicase-mediated mitochondrial reactive oxygen species production in *Arabidopsis* mediates crosstalk between abscisic acid and auxin signaling. *Plant Cell* 24 1815–1833. 10.1105/tpc.112.098707 22652060 PMC3442571

[B43] Hill R. D. (2012). Non-symbiotic haemoglobins-What’s happening beyond nitric oxide scavenging? *AoB Plants* 2012:pls004 10.1093/aobpla/pls004 PMC3292739 22479675

[B44] Holman T. J. Jones P. D. Russell L. Medhurst A. Tomas S. U. Talloji P. (2009). The N-end rule pathway promotes seed germination and establishment through removal of ABA sensitivity in *Arabidopsis*. *Proc. Natl. Acad. Sci. U.S.A.* 106 4549–4554. 10.1073/pnas.0810280106 19255443 PMC2649959

[B45] Hongay C. F. Grisafi P. L. Galitski T. Fink G. R. (2006). Antisense transcription controls cell fate in *Saccharomyces cerevisiae*. *Cell* 127 735–745. 10.1016/j.cell.2006.09.038 17110333

[B46] Hsieh T. F. Shin J. Uzawa R. Silva P. Cohen S. Bauer M. J. (2011). Regulation of imprinted gene expression in *Arabidopsis* endosperm. *Proc. Natl. Acad. Sci. U.S.A.* 108 1755–1762. 10.1073/pnas.1019273108 21257907 PMC3033266

[B47] Hsueh Y. L. Lou C. H. (1947). Effects of 2,4-D on seed germination and respiration. *Science* 105 283–285. 10.1126/science.105.2724.283 17835144

[B48] Ikeda Y. Kinoshita T. (2009). DNA demethylation: a lesson from the garden. *Chromosoma* 118 37–41. 10.1007/s00412-008-0183-3 18839198

[B49] Jiang Z. M. Xu G. Jing Y. J. Tang W. J. Lin R. C. (2016). Phytochrome B and REVEILLE1/2-mediated signalling controls seed dormancy and germination in *Arabidopsis*. *Nat. Commun.* 7:12377 10.1038/ncomms12377 PMC4987513 27506149

[B50] Jones R. L. Armstrong J. E. (1971). Evidence for osmotic regulation of hydrolytic enzyme production in germinating barley seeds. *Plant Physiol.* 48 137–142. 10.1104/pp.48.2.137 16657750 PMC396818

[B51] Jullien P. E. Berger F. (2009). Gamete-specific epigenetic mechanisms shape genomic imprinting. *Curr. Opin. Plant Biol.* 12 637–642. 10.1016/j.pbi.2009.07.004 19709923

[B52] Kang J. Hwang J. U. Lee M. Kim Y. Y. Assmann S. M. Martinoia E. (2010). PDR-type ABC transporter mediates cellular uptake of the phytohormone abscisic acid. *Proc. Natl. Acad. Sci. U.S.A.* 107 2355–2360. 10.1073/pnas.0909222107 20133880 PMC2836657

[B53] Kang J. Yim S. Choi H. Kim A. Lee K. P. Lopez-Molina L. (2015). Abscisic acid transporters cooperate to control seed germination. *Nat. Commun.* 6:8113 10.1038/ncomms9113 PMC4569717 26334616

[B54] Kanno Y. Hanada A. Chiba Y. Ichikawa T. Nakazawa M. Matsui M. (2012). Identification of an abscisic acid transporter by functional screening using the receptor complex as a sensor. *Proc. Natl. Acad. Sci. U.S.A.* 109 9653–9658. 10.1073/pnas.1203567109 22645333 PMC3386071

[B55] Kerscher O. Felberbaum R. Hochstrasser M. (2006). Modification of proteins by ubiquitin and ubiquitin-like proteins. *Annu. Rev. Cell Dev. Biol.* 22 159–180. 10.1146/annurev.cellbio.22.010605.093503 16753028

[B56] Kinoshita T. Miura A. Choi Y. H. Kinoshita Y. Cao X. F. Jacobsen S. E. (2004). One-way control of *FWA* imprinting in *Arabidopsis* endosperm by DNA methylation. *Science* 303 521–523. 10.1126/science.1089835 14631047

[B57] Konishi M. Yanagisawa S. (2010). Identification of a nitrate-responsive *cis*-element in the Arabidopsis *NIR1* promoter defines the presence of multiple *cis*-regulatory elements for nitrogen response. *Plant J.* 63 269–282. 10.1111/j.1365-313X.2010.04239.x 20444232

[B58] Konishi M. Yanagisawa S. (2011). The regulatory region controlling the nitrate-responsive expression of a nitrate reductase gene, NIA1, in Arabidopsis. *Plant Cell Physiol.* 52 824–836. 10.1093/pcp/pcr033 21454300

[B59] Konishi M. Yanagisawa S. (2013). Arabidopsis NIN-like transcription factors have a central role in nitrate signalling. *Nat. Commun.* 4 1617 10.1038/ncomms2621 23511481

[B60] Kornienko A. E. Guenzl P. M. Barlow D. P. Pauler F. M. (2013). Gene regulation by the act of long non-coding RNA transcription. *BMC Biol.* 11:59 10.1186/1741-7007-11-59 PMC3668284 23721193

[B61] Kuromori T. Miyaji T. Yabuuchi H. Shimizu H. Sugimoto E. Kamiya A. (2010). ABC transporter AtABCG25 is involved in abscisic acid transport and responses. *Proc. Natl. Acad. Sci. U.S.A.* 107 2361–2366. 10.1073/pnas.0912516107 20133881 PMC2836683

[B62] Kuromori T. Sugimoto E. Shinozaki K. (2014). Intertissue signal transfer of abscisic acid from vascular cells to guard cells. *Plant Physiol.* 164 1587–1592. 10.1104/pp.114.235556 24521878 PMC3982725

[B63] Kushiro T. Okamoto M. Nakabayashi K. Yamagishi K. Kitamura S. Asami T. (2004). The *Arabidopsis* cytochrome P450 CYP707A encodes ABA 8’-hydroxylases: key enzymes in ABA catabolism. *EMBO J.* 23 1647–1656. 10.1038/sj.emboj.7600121 15044947 PMC391058

[B64] Lee N. Choi G. (2017). Phytochrome-interacting factor from *Arabidopsis* to liverwort. *Curr. Opin. Plant Biol.* 35 54–60. 10.1016/j.pbi.2016.11.004 27875778

[B65] Leran S. Varala K. Boyer J. C. Chiurazzi M. Crawford N. Daniel-Vedele F. (2014). A unified nomenclature of NITRATE TRANSPORTER 1/PEPTIDE TRANSPORTER family members in plants. *Trends Plant Sci.* 19 5–9. 10.1016/j.tplants.2013.08.008 24055139

[B66] Lim S. Park J. Lee N. Jeong J. Toh S. Watanabe A. (2013). ABA-INSENSITIVE3, ABA-INSENSITIVE5, and DELLAs interact to activate the expression of *SOMNUS* and other high-temperature-inducible genes in imbibed seeds in *Arabidopsis*. *Plant Cell* 25 4863–4878. 10.1105/tpc.113 24326588 PMC3903992

[B67] Liu H. Stone S. L. (2014). Regulation of ABI5 turnover by reversible post-translational modifications. *Plant Signal. Behav.* 9:e27577 10.4161/psb.27577 PMC4091339 24398698

[B68] Liu H. X. Stone S. L. (2010). Abscisic acid increases *Arabidopsis* ABI5 transcription factor levels by promoting KEG E3 ligase self-ubiquitination and proteasomal degradation. *Plant Cell* 22 2630–2641. 10.1105/tpc.110.076075 20682837 PMC2947163

[B69] Liu P.-P. Montgomery T. A. Fahlgren N. Kasschau K. D. Nonogaki H. Carrington J. C. (2007). Repression of AUXIN RESPONSE FACTOR10 by microRNA160 is critical for seed germination and post-germination stages. *Plant J.* 52 133–146. 10.1111/j.1365-313X.2007.03218.x 17672844

[B70] Liu Y. Geyer R. van Zanten M. Carles A. Li Y. Hörold A. (2011). Identification of the *Arabidopsis REDUCED DORMANCY 2* gene uncovers a role for the polymerase associated factor 1 complex in seed dormancy. *PLoS ONE* 6:e22241 10.1371/journal.pone.0022241 PMC3143138 21799800

[B71] Liu Y. G. Shi L. Ye N. H. Liu R. Jia W. S. Zhang J. H. (2009). Nitric oxide-induced rapid decrease of abscisic acid concentration is required in breaking seed dormancy in Arabidopsis. *New Phytol.* 183 1030–1042. 10.1111/j.1469-8137.2009.02899.x 19522839

[B72] Liu Y. Koornneef M. Soppe W. J. (2007). The absence of histone H2B monoubiquitination in the *Arabidopsis hub1* (*rdo4*) mutant reveals a role for chromatin remodeling in seed dormancy. *Plant Cell* 19 433–444. 10.1105/tpc.106.049221 17329563 PMC1867323

[B73] Lopez-Molina L. Mongrand S. Chua N.-H. (2001). A postgermination developmental arrest checkpoint is mediated by abscisic acid and requires the ABI5 transcription factor in *Arabidopsis*. *Proc. Natl. Acad. Sci. U.S.A.* 98 4782–4787. 10.1073/pnas.081594298 11287670 PMC31911

[B74] Lotan T. Ohto M.-A. Yee K. M. West M. A. L. Lo R. Kwong R. W. (1998). *Arabidopsis* LEAFY COTYLEDON1 is sufficient to induce embryo development in vegetative cells. *Cell* 93 1195–1205. 10.1016/S0092-8674(00)81463-4 9657152

[B75] Maher E. P. Martindale S. J. B. (1980). Mutants of *Arabidopsis thaliana* with altered responses to auxins and gravity. *Biochem. Genet.* 18 1041–1053. 10.1007/BF00484337 7247923

[B76] Martinez-Andujar C. Pluskota W. E. Bassel G. W. Asahina M. Pupel P. Nguyen T. T. (2012). Mechanisms of hormonal regulation of endosperm cap-specific gene expression in tomato seeds. *Plant J.* 71 575–586. 10.1111/j.1365-313X.2012.05010.x 22458548

[B77] Masubelele N. H. Dewitte W. Menges M. Maughan S. Collins C. Huntley R. (2005). D-type cyclins activate division in the root apex to promote seed germination in *Arabidopsis*. *Proc. Natl. Acad. Sci. U.S.A.* 102 15694–15699. 10.1073/pnas.0507581102 16227434 PMC1266134

[B78] Matakiadis T. Alboresi A. Jikumaru Y. Tatematsu K. Pichon O. Renou J.-P. (2009). The Arabidopsis abscisic acid catabolic gene *CYP707A2* plays a key role in nitrate control of seed dormancy. *Plant Physiol.* 149 949–960. 10.1104/pp.108.126938 19074630 PMC2633832

[B79] McGinnis K. M. Thomas S. G. Soule J. D. Strader L. C. Zale J. M. Sun T. P. (2003). The Arabidopsis *SLEEPY1* gene encodes a putative F-box subunit of an SCF E3 ubiquitin ligase. *Plant Cell* 15 1120–1130. 10.1105/tpc.010827 12724538 PMC153720

[B80] McKibbin R. S. Wilkinson M. D. Bailey P. C. Flintham J. E. Andrew L. M. Lazzeri P. A. (2002). Transcripts of *Vp-1* homeologues are misspliced in modern wheat and ancestral species. *Proc. Natl. Acad. Sci. U.S.A.* 99 10203–10208. 10.1073/pnas.152318599 12119408 PMC126648

[B81] Miura K. Jin J. B. Hasegawa P. M. (2007). Sumoylation, a post-translational regulatory-process in plants. *Curr. Opin. Plant Biol.* 10 495–502. 10.1016/j.pbi.2007.07.002 17720613

[B82] Miura K. Lee J. Jin J. B. Yoo C. Y. Miura T. Hasegawa P. M. (2009). Sumoylation of ABI5 by the *Arabidopsis* SUMO E3 ligase SIZ1 negatively regulates abscisic acid signaling. *Proc. Natl. Acad. Sci. U.S.A.* 106 5418–5423. 10.1073/pnas.0811088106 19276109 PMC2664011

[B83] Moore T. Haig D. (1991). Genomic imprinting in mammalian development: a parental tug-of-war. *Trends Genet.* 7 45–49. 10.1016/0168-9525(91)90230-N 2035190

[B84] Mortensen S. A. Grasser K. D. (2014). The seed dormancy defect of *Arabidopsis* mutants lacking the transcript elongation factor TFIIS is caused by reduced expression of the *DOG1* gene. *FEBS Lett.* 588 47–51. 10.1016/j.febslet.2013.10.047 24252221

[B85] Nakabayashi K. Bartsch M. Ding J. Soppe W. J. (2015). Seed dormancy in Arabidopsis requires self-binding ability of DOG1 protein and the presence of multiple isoforms generated by alternative splicing. *PLoS Genet.* 11:e1005737 10.1371/journal.pgen.1005737 PMC4686169 26684465

[B86] Nakabayashi K. Bartsch M. Xiang Y. Miatton E. Pellengahr S. Yano R. (2012). The time required for dormancy release in *Arabidopsis* is determined by DELAY OF GERMINATION1 protein levels in freshly harvested seeds. *Plant Cell* 24 2826–2838. 10.1105/tpc.112.100214 22829147 PMC3426117

[B87] Nakamura S. Pourkheirandish M. Morishige H. Kubo Y. Nakamura M. Ichimura K. (2016). Mitogen-activated protein kinase kinase 3 regulates seed dormancy in barley. *Curr. Biol.* 26 775–781. 10.1016/j.cub.2016.01.024 26948880

[B88] Nee G. Xiang Y. Soppe W. J. (2016). The release of dormancy, a wake-up call for seeds to germinate. *Curr. Opin. Plant Biol.* 35 8–14. 10.1016/j.pbi.2016.09.002 27710774

[B89] Nonogaki H. (2010). MicroRNA gene regulation cascades during early stages of plant development. *Plant Cell Physiol.* 51 1840–1846. 10.1093/pcp/pcq154 20937608

[B90] Nonogaki H. (2014). Seed dormancy and germination-emerging mechanisms and new hypotheses. *Front. Plant Sci.* 5:233 10.3389/fpls.2014.00233 PMC4036127 24904627

[B91] Nonogaki H. Gee O. H. Bradford K. J. (2000). A germination-specific endo-beta-mannanase gene is expressed in the micropylar endosperm cap of tomato seeds. *Plant Physiol.* 123 1235–1246. 10.1104/pp.123.4.1235 10938343 PMC59083

[B92] Nonogaki M. Sekine T. Nonogaki H. (2015). Chemically inducible gene expression in seeds before testa rupture. *Seed Sci. Res.* 25 345–352. 10.1017/S0960258515000240

[B93] Ohto M. Stone S. L. Harada J. J. (2007). “Genetic control of seed development and seed mass,” in *Seed Development, Dormancy and Germination* eds Bradford K. J. Nonogaki H. (Oxford: Blackwell Publishing) 1–24. 10.1002/9780470988848

[B94] Okamoto M. Kuwahara A. Seo M. Kushiro T. Asami T. Hirai N. (2006). CYP707A1 and CYP707A2, which encode abscisic acid 8 ’-hydroxylases, are indispensable for proper control of seed dormancy and germination in Arabidopsis. *Plant Physiol.* 141 97–107. 10.1104/pp.106.079475 16543410 PMC1459320

[B95] Park K. Kim M. Y. Vickers M. Park J. S. Hyun Y. Okamoto T. (2016). DNA demethylation is initiated in the central cells of *Arabidopsis* and rice. *Proc. Natl. Acad. Sci. U.S.A.* 113 15138–15143. 10.1073/pnas.1619047114 27956642 PMC5206524

[B96] Park Y. Xu Z. Y. Kim S. Y. Lee J. Choi B. Lee J. (2016). Spatial regulation of ABCG25, an ABA exporter, is an important component of the mechanism controlling cellular ABA levels. *Plant Cell* 28 2528–2544. 10.1105/tpc.16.00359 27697789 PMC5134978

[B97] Pelechano V. Steinmetz L. M. (2013). Non-coding RNA gene regulation by antisense transcription. *Nat. Rev. Genet.* 14 880–893. 10.1038/nrg3594 24217315

[B98] Penterman J. Zilberman D. Huh J. H. Ballinger T. Henikoff S. Fischer R. L. (2007). DNA demethylation in the *Arabidopsis* genome. *Proc. Natl. Acad. Sci. U.S.A.* 104 6752–6757. 10.1073/pnas.0701861104 17409185 PMC1847597

[B99] Perazzolli M. Dominici P. Romero-Puertas M. C. Zago E. Zeier A. Sonoda M. (2004). Arabidopsis nonsymbiotic hemoglobin AHb1 modulates nitric oxide bioactivity. *Plant Cell* 16 2785–2794. 10.1105/tpc.104.025379 15367716 PMC520971

[B100] Pignatta D. Gehring M. (2012). Imprinting meets genomics: new insights and new challenges. *Curr. Opin. Plant Biol.* 15 530–535. 10.1016/j.pbi.2012.09.004 23000433

[B101] Piskurewicz U. Iwasaki M. Susaki D. Megies C. Kinoshita T. Lopez-Molina L. (2016). Dormancy-specific imprinting underlies maternal inheritance of seed dormancy in *Arabidopsis thaliana*. *eLife* 5:e19573 10.7554/eLife.19573 PMC5243116 28005006

[B102] Piskurewicz U. Jikumaru Y. Kinoshita N. Nambara E. Kamiya Y. Lopez-Molina L. (2008). The gibberellic acid signaling repressor RGL2 inhibits *Arabidopsis* seed germination by stimulating abscisic acid synthesis and ABI5 activity. *Plant Cell* 20 2729–2745. 10.1105/tpc.108.061515 18941053 PMC2590721

[B103] Quinn J. J. Chang H. Y. (2016). Unique features of long non-coding RNA biogenesis and function. *Nat. Rev. Genet.* 17 47–62. 10.1038/nrg.2015.10 26666209

[B104] Ragni L. Nieminen K. Pacheco-Villalobos D. Sibout R. Schwechheimer C. Hardtke C. S. (2011). Mobile gibberellin directly stimulates *Arabidopsis* hypocotyl xylem expansion. *Plant Cell* 23 1322–1336. 10.1105/tpc.111.084020 21498678 PMC3101547

[B105] Regnault T. Davière J.-M. Wild M. Sakvarelidze-Achard L. Heintz D. Carrera Bergua E. (2015). The gibberellin precursor GA12 acts as a long-distance growth signal in Arabidopsis. *Nat. Plants* 1:15073 10.1038/nplants.2015.73 27250008

[B106] Reyes J. L. Chua N.-H. (2007). ABA induction of miR159 controls transcript levels of two MYB factors during Arabidopsis seed germination. *Plant J.* 49 592–606. 10.1111/j.1365-313X.2006.02980.x 17217461

[B107] Sato K. Yamane M. Yamaji N. Kanamori H. Tagiri A. Schwerdt J. G. (2016). Alanine aminotransferase controls seed dormancy in barley. *Nat. Commun.* 7:11625 10.1038/ncomms11625 PMC4873977 27188711

[B108] Scott R. J. Spielman M. Bailey J. Dickinson H. G. (1998). Parent-of-origin effects on seed development in *Arabidopsis thaliana*. *Development* 125 3329–3341. 9693137 10.1242/dev.125.17.3329

[B109] Seo M. Nambara E. Choi G. Yamaguchi S. (2009). Interaction of light and hormone signals in germinating seeds. *Plant Mol. Biol.* 69 463–472. 10.1007/s11103-008-9429-y 19031046

[B110] Shani E. Weinstain R. Zhang Y. Castillejo C. Kaiserli E. Chory J. (2013). Gibberellins accumulate in the elongating endodermal cells of *Arabidopsis* root. *Proc. Natl. Acad. Sci. U.S.A.* 110 4834–4839. 10.1073/pnas.1300436110 23382232 PMC3606980

[B111] Shearwin K. E. Callen B. P. Egan J. B. (2005). Transcriptional interference - a crash course. *Trends Genet.* 21 339–345. 10.1016/j.tig.2005.04.009 15922833 PMC2941638

[B112] Stone S. Williams L. Farmer L. Vierstra R. Callis J. (2006). KEEP ON GOING, a RING E3 ligase essential for *Arabidopsis* growth and development, is involved in abscisic acid signaling. *Plant Cell* 18 3415–3428. 10.1105/tpc.106.046532 17194765 PMC1785414

[B113] Sugliani M. Brambilla V. Clerkx E. J. M. Koornneef M. Soppe W. J. J. (2010). The conserved splicing factor SUA controls alternative splicing of the developmental regulator *ABI3* in *Arabidopsis*. *Plant Cell* 22 1936–1946. 10.1105/tpc.110.074674 20525852 PMC2910958

[B114] Tal I. Zhang Y. Jorgensen M. E. Pisanty O. Barbosa I. C. R. Zourelidou M. (2016). The *Arabidopsis* NPF3 protein is a GA transporter. *Nat. Commun.* 7:11486 10.1038/ncomms11486 PMC4857387 27139299

[B115] Tasaki T. Kwon Y. T. (2007). The mammalian N-end rule pathway: new insights into its components and physiological roles. *Trends Biochem. Sci.* 32 520–528. 10.1016/j.tibs.2007.08.010 17962019

[B116] Tasaki T. Sriram S. M. Park K. S. Kwon Y. T. (2012). The N-end rule pathway. *Annu. Rev. Biochem.* 81 261–289. 10.1146/annurev-biochem-051710-093308 22524314 PMC3610525

[B117] Toh S. Imamura A. Watanabe A. Nakabayashi K. Okamoto M. Jikumaru Y. (2008). High temperature-induced abscisic acid biosynthesis and its role in the inhibition of gibberellin action in Arabidopsis seeds. *Plant Physiol.* 146 1368–1385. 10.1104/pp.107.113738 18162586 PMC2259091

[B118] Tonosaki K. Kinoshita T. (2015). Possible roles for polycomb repressive complex 2 in cereal endosperm. *Front. Plant Sci.* 6:144 10.3389/fpls.2015.00144 PMC4357243 25814998

[B119] Torada A. Koike M. Ogawa T. Takenouchi Y. Tadamura K. Wu J. (2016). A causal gene for seed dormancy on wheat chromosome 4A encodes a MAP kinase kinase. *Curr. Biol.* 26 782–787. 10.1016/j.cub.2016.01.063 26948878

[B120] Ueguchi-Tanaka M. Ashikari M. Nakajima M. Itoh H. Katoh E. Kobayashi M. (2005). *GIBBERELLIN INSENSITIVE DWARF1* encodes a soluble receptor for gibberellin. *Nature* 437 693–698. 10.1038/nature04028 16193045

[B121] Ueguchi-Tanaka M. Nakajima M. Motoyuki A. Matsuoka M. (2007). Gibberellin receptor and its role in gibberellin signaling in plants. *Annu. Rev. Plant Biol.* 58 183–198. 10.1146/annurev.arplant.58.032806.103830 17472566

[B122] Wang P. Zhu J.-K. Lang Z. (2015). Nitric oxide suppresses the inhibitory effect of abscisic acid on seed germination by S-nitrosylation of SnRK2 proteins. *Plant Signal. Behav.* 10:e1031939 10.1080/15592324.2015.1031939 PMC4622540 26024299

[B123] Wang Z. Cao H. Sun Y. Li X. Chen F. Carles A. (2013). *Arabidopsis* paired amphipathic helix proteins SNL1 and SNL2 redundantly regulate primary seed dormancy via abscisic acid-ethylene antagonism mediated by histone deacetylation. *Plant Cell* 25 149–166. 10.1105/tpc.112.108191 23371947 PMC3584531

[B124] Wang Z. Chen F. Y. Li X. Y. Cao H. Ding M. Zhang C. (2016). Arabidopsis seed germination speed is controlled by SNL histone deacetylase-binding factor-mediated regulation of AUX1. *Nat. Commun.* 7:13412 10.1038/ncomms13412 PMC5114640 27834370

[B125] West M. Harada J. J. (1993). Embryogenesis in higher plants: an overview. *Plant Cell* 5 1361–1369. 10.1105/tpc.5.10.1361 12271035 PMC160368

[B126] Yamaguchi S. Kamiya Y. Sun T. (2001). Distinct cell-specific expression patterns of early and late gibberellin biosynthetic genes during *Arabidopsis* seed germination. *Plant J.* 28 443–453. 10.1046/j.1365-313X.2001.01168.x 11737781

[B127] Yan D. Easwaran V. Chau V. Okamoto M. Ierullo M. Kimura M. (2016). NIN-like protein 8 is a master regulator of nitrate-promoted seed germination in *Arabidopsis*. *Nat. Commun.* 7:13179 10.1038/ncomms13179 PMC5064020 27731416

[B128] Yang Y. D. Hammes U. Z. Taylor C. G. Schachtman D. P. Nielsen E. (2006). High-affinity auxin transport by the AUX1 influx carrier protein. *Curr. Biol.* 16 1123–1127. 10.1016/j.cub.2006.04.029 16677815

[B129] Yano R. Takebayashi Y. Nambara E. Kamiya Y. Seo M. (2013). Combining association mapping and transcriptomics identify *HD2B* histone deacetylase as a genetic factor associated with seed dormancy in *Arabidopsis thaliana*. *Plant J.* 74 815–828. 10.1111/tpj.12167 23464703

[B130] Zhu A. Greaves I. K. Dennis E. S. Peacock W. J. (2017). Genome-wide analyses of four major histone modifications in Arabidopsis hybrids at the germinating seed stage. *BMC Genomics* 18:137 10.1186/s12864-017-3542-8 PMC5297046 28173754

[B131] Zhu J. Kapoor A. Sridhar V. V. Agius F. Zhu J.-K. (2007). The DNA glycosylase/lyase ROS1 functions in pruning DNA methylation patterns in *Arabidopsis*. *Curr. Biol.* 17 54–59. 10.1016/j.cub.2006.10.059 17208187

